# Early supported hospital discharge for foot disease: a co-design study

**DOI:** 10.1186/s12913-021-06925-z

**Published:** 2021-10-15

**Authors:** Rebecca Jessup, Samantha Hanna, Jaspreet Kaur, Iman Bayat, Cassandra Bramston

**Affiliations:** 1 Department of Education and Research, Northern Health, 185 Cooper Street, Epping, Victoria 3076 Australia; 2 Podiatry and Orthotics Department, Austin Health, 145 Studley Road, Heidelberg, Victoria 3084 Australia; 3 Podiatry and Orthotics Department, Northern Health, 185 Cooper Street, Epping, Victoria 3076 Australia; 4 Vascular Department, Northern Health, 185 Cooper Street, Epping, Victoria 3076 Australia

**Keywords:** Foot disease, Peripheral vascular disease

## Abstract

**Background:**

There are more than 10,000 admissions each year in Australia for foot disease, with an average length of hospital stay of 26 days. Early supported discharge (ESD) has been shown to improve patient satisfaction and reduce length of stay without increasing the risk of 30-day readmissions. This research aims to gain consensus on an optimal model of early supported discharge for foot disease.

**Methods:**

Three focus groups were held where preliminary components for an early discharge model, as well as inclusion and exclusion criteria, were identified with a purposefully sampled group of medical, nursing, allied health staff and consumers. Two researchers independently systematically coded focus group transcripts to identify components of an ESD model using an iterative constant comparative method. These components then formed the basis of a three phase Delphi study, with all individuals from the focus groups were invited to act as panellists. Panellists rated components for their importance with consensus established as a rating of either essential or very important by ≥80% of the panel.

**Results:**

Twenty-nine experts (including 5 consumers) participated across the two study phases. Twenty-three (3 consumers) participated in the focus groups in phase one. Twenty-eight of the twenty-nine experts participated in the phase 2 Delphi. 21/28 completed round 1 of the Delphi (75% response rate), 22/28 completed round 2 (79% response rate), and 16/22 completed round 3 (72% response rate). Consensus was achieved for 17 (29%) of 58 components. These included changes to the way patients are managed on wards (both location and timeliness of care by the multidisciplinary team) and the addition of new workforce roles to improve co-ordination and management of the patients once they are at home.

**Conclusions:**

A model of early supported discharge that would allow individuals to return home earlier in a way that is safe, acceptable, and feasible may result in improving patient satisfaction while reducing health system burden. Future trial and implementation of the ESD model identified in this study has the potential to make a significant contribution to the experience of care for patients and to the sustainability of the health system.

## Background

Foot disease, including ulcers, infection, and lower limb ischaemia is a leading cause of hospitalisation in Australia, accounting for approximately 5% of hospitalisations [1]. There are more than 10,000 admissions each year in Australia for diabetes-related foot disease, with an average length of hospital stay of 26 days [2, 3]. Recently published research suggests affected individuals experience very high rates of unplanned 30 day readmissions (around 17%) [4]. A recent cost-effectiveness analysis on care for patients with diabetic foot disease found that provision of care in an optimal way results in both clinically important health benefits measured in quality-adjusted life years (QALYs) and overall cost savings for high-risk patients when compared with usual care [5]. QALYs and costs savings ranged between 0.13 and $9100.11 respectively for those aged 35–54 years, to 0.16 and $12,394.97 respectively for those aged 75 years or older [5].

Early supported discharge (ESD) models allow patients to return home earlier than usual by replacing some of their hospital treatment with care in the home environment. ESD models have been trialled for a number of different conditions including stroke [6], acute exacerbations of COPD [7], hip and knee replacements and other types of orthopaedic conditions in elderly patients [8–11]. Some of the purported benefits of providing ESD in these studies have included improvements in shared decision making, improved patient motivation through focusing on realistic rehabilitation goals, provision of contextually relevant education, treatment and rehabilitation, increased focus on self-directed activities, and fostering a more realistic understanding of recovery [6, 7].

Co-design is a participatory approach to the development of interventions that brings together employee and user experience to design a mutually agreed upon solution [12]. In health care, the approach brings together clinicians and patients to negotiate expectations with the aim of creating solutions that are sensitive to the local context [13]. In this way, co-design harnesses the collective creativity and tacit knowledge of clinicians *and* consumer to create solutions that are acceptable to both groups and therefore more likely to be adopted and sustained in the long term [13].

The aim of this study was to use a process of co-design to identify and then gain consensus on the key components of an optimal model of early supported discharge for individuals who are hospitalised with foot disease.

## Methods

### Design

A two phase sequential qualitative design was employed in this study:

- Phase one: consisted of focus groups with consumers and clinicians with the aim of generating components of an ideal model of ESD for foot disease; and
- Phase two consisted of a three round Delphi survey [14, 15] that aimed to gained consensus on the components that should be included in an ideal model of ESD for foot disease.

### Setting

Northern Health (NH) is the major provider of acute, sub-acute and ambulatory specialist services in Melbourne’s north. It provides comprehensive primary, secondary and tertiary healthcare services. Residents in the NH catchment area are ethnically and culturally diverse and originate from more than 165 countries and speak more than 100 languages. The area has lower levels of income, educational attainment and health literacy and higher rates of unemployment than Victorian state averages [16–18].

Currently, patients admitted with foot disease at the hospital may be admitted on a number of different wards, and may be admitted under vascular, endocrinology or infectious diseases bed cards, depending on the primary diagnosis on admission. Patients may be admitted on a planned admission, but most often they are admitted following presentation to the emergency department. Over the past 5 years the hospital has worked hard to improve the fragmentation of services for patients admitted with foot disease and most patients are now cared for by a multidisciplinary team consisting of vascular, podiatry, infectious diseases, orthotics, dietetics, occupational therapy and physiotherapy. Most patients undergo surgical revascularisation and/ or amputation and/ or debridement during their hospital stay, followed by a period of in hospital treatment with antibiotics, dressings and implementation of offloading (use of such as orthotics that reduce pressure at the site of ulceration) strategies.

### Participants

For phase one we employed purposeful sampling to identify clinicians with recognised expertise in the treatment and management of foot disease, with additional snowballing technique (invited participants could suggest additional participants). Patient participants were purposefully sampled from individuals who were over the age of 18 years and who had experienced one or more hospital admissions for foot disease in the previous 12 months. The clinician focus groups were held separately to the consumer focus groups to ensure the consumers felt comfortable and confident to speak up about their needs.

For phase two (Delphi study) we invited all participants who were invited to participate in the focus groups, independent of whether they had been able to attend. We allowed new participants to join in round 2 if they had been unable to participate in round 1 (as round 1 was not a scoring round), however no new participants were allowed in round 3 (final round). The Dillman tailored design method was used to invite clinicians [19], with the initial invitation sent via a personalised email with a link to the survey, with up to three additional reminder email reminders sent at weekly intervals. For consumers, the Delphi surveys were completed during outpatient follow up appointments. The patient participants were supported to complete the surveys by a research assistant who provided a description of each component in layman terms to help them with their understanding. We permitted patient participants to also be assisted by family or an interpreter as required. We established a participation rate of at least 70% would ensure credibility and validity of the results [14, 20].

### Data collection

**Fig. 1:**
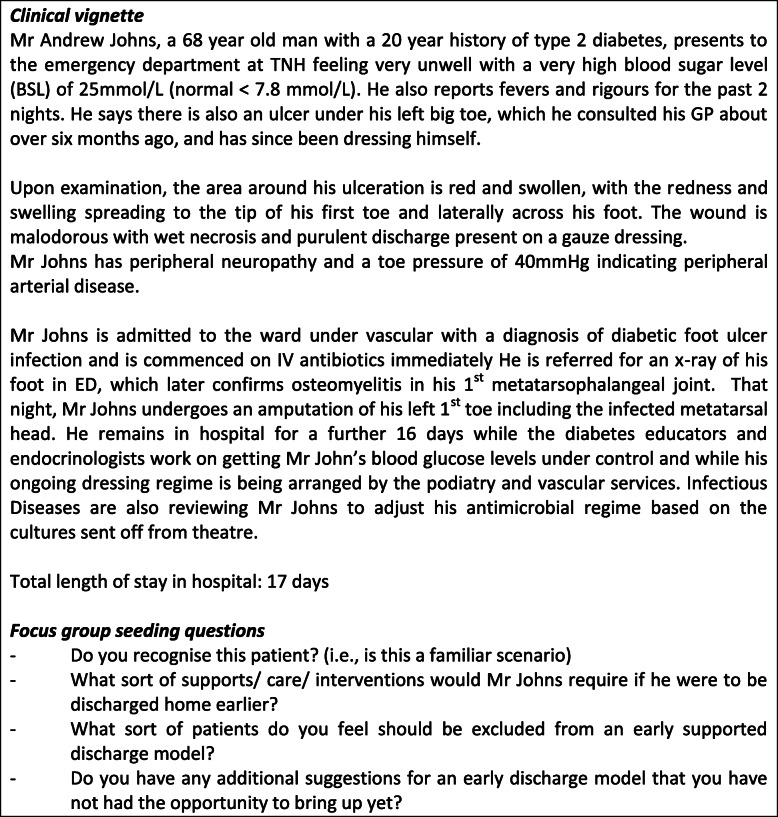
Example of clinical vignette and seeding questions used in the staff focus groups

Three focus groups were conducted in phase one, one group with consumers and two with clinicians. We aimed for 6 to 12 participants per focus group based on the optimum number to ensure broad representation across disciplines and experience while also ensuring enough opportunity for all participants to share their insights and observations [21]. At each focus group a clinical case scenario of a ‘typical’ (fictional) patient was presented and attendees were invited to suggest solutions to support an early discharge to home and prevention of unplanned hospital readmissions. Figure 1 provides an example of the clinical vignette used for the staff focus groups, as well as the seeding questions used to generate discussion. A modified version of this same scenario was used for the patient focus group, however all health care terminology was replaced with lay terms.

**Fig. 2:**
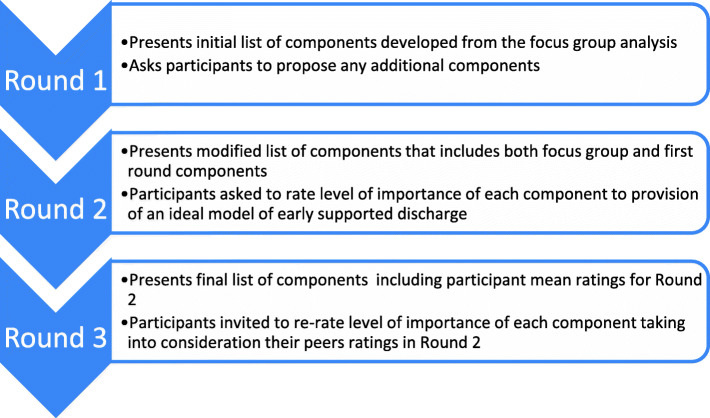
Three round Delphi process

Phase two of the study consisted of a three round Delphi survey (Fig. 2). The first round comprised two parts: part one included questions relating to respondents characteristics (for staff this included current role in the organisation, for consumers this consisted of age and gender only). Part two asked each participant to review components of the model identified in the focus groups in phase one, and provide additional potential components (if they felt any were missing) with a brief justification. This round also acted as a form of member checking for the themes arising from the focus groups in phase one.

**Fig. 3:**
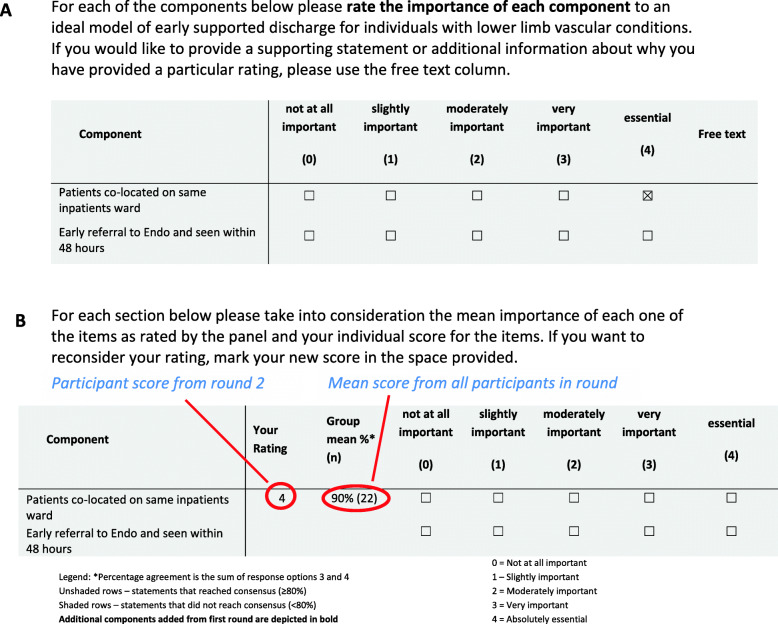
Example of second (**A**) and third (**B**) rounds of the Delphi Survey

The second and third rounds were both scoring rounds. In the second round, respondents were asked to complete a questionnaire based on the information provided in the first round (i.e. a list of original components of the model as well as any additional components proposed in the first round) (Fig. 3, A). Respondents were invited to score how important they felt each component of the model was to providing an ‘ideal’ model of early supported discharge on a 5-point Likert scale (not at all important through to essential). Participants were provided with a free text section for supporting comments.

In the third and final survey round, participants were provided with the results of the second round in the form of a mean group response and any qualifying (free text) statements. They were asked to re-score the components, taking into consideration the mean importance of each as rated by the panel in the previous round (Fig. 3, B). The top two highest categories on the Likert scale (very important and essential) were considered to be agreement from a participant that the component should form part of the final model.

### Data analysis

Two researchers (RJ and JK) independently and systematically coded the focus group transcripts from phase one using an iterative constant comparative method [22]. The process involved two stages – initial coding to gain the broadest range of themes, followed by focused coding to reduce overlap and redundancy of coding [23]. Two cross-checks to compare emerging themes was performed to ensure accuracy of inferences. Where conflicts arose between coders and agreement could not be reached a third reviewer (SH) was consulted. A pragmatic approach was adopted when drawing final inferences from the transcripts, with a focus on the development of useful knowledge directly related to an optimal model of early discharge. Final inferences were verified by subject specialists (IB and SH). Most importantly, and consistent with the co-design process, the data was then referred back to those who attended the workshops (final respondent validation) in the first round of the Delphi survey.

Achieving consensus in the Delphi was defined as ≥80% of the respondents in agreement on the value of a given component, with agreement being set as the two highest scale response options (very important and essential) [24]. We aimed for a 70% response rate in each round to ensure validity of results [20].

### Ethics approval and informed consent to participate

Ethics approval was obtained by the Northern Health Low Risk Ethics Committee, reference number 55150. All staff and consumers provided written informed consent prior to participation in the research. Ethics approval was provided by the Northern Health LREC and consent to written informed consent to participate was gained from all participants in accordance with the Australian National Statement on Ethical Conduct in Human Research 2007 and the Australian Code for Responsible Conduct of Research 2007.

## Results

In total, 5 consumers and 24 clinicians from medical, nursing, and allied health disciplines with experience working with patients with foot disease were approached to participate in the study. In phase one, 20 clinicians (87% participation) and 3 consumers (60% participation) participated in the three focus groups. A total of 32 components were identified during the focus groups coding.

All 28 of the 29 participants approached participated in the Delphi panel. Table 1 provides an overview of participant expertise. Sixteen staff and five consumers contributed to the first round (75% response rate), 17 staff and five consumers contributed to the second round (79% response rate), and 13 staff along with three consumers contributed to the third round (72% response rate of eligible participants from round two).

**Table 1:** Focus group and Delphi Panel Expertise (*n* = 29)

***Consumer participants***	5 (80% male)
***Allied Health Participants***	
Occupational therapist	1
Orthotist	1
Physiotherapist	1
Podiatrist	2
***Medical Participants***	
Endocrinologist	2
Geriatrician	1
Infectious Diseases Specialist	1
Orthopaedic surgeon	1
Vascular surgeon	5
***Nursing Participants***	
Diabetes Educator	1
Registered nurse (hospital in the home and wound clinics)	7
Wound nurse consultant	1
Patients	5

**Fig. 4:**
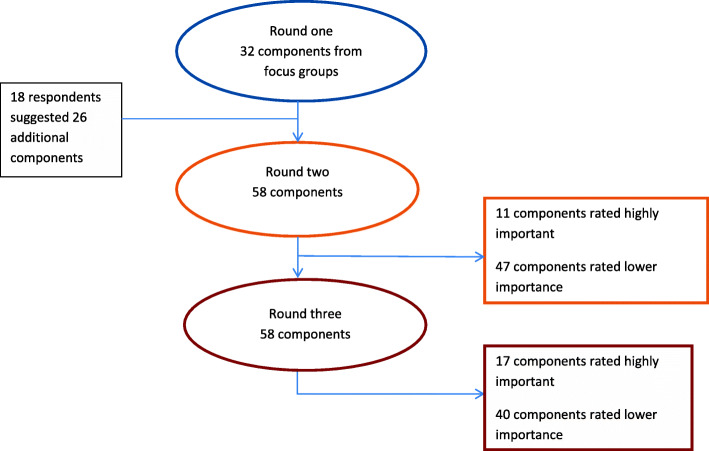
Results of the three round Delphi survey

Figure 4 provides an overview of the results of the three rounds. From the initial 32 items identified from the focus groups, an additional 26 components were added in the first round of the Delphi. In the second round, only 11 components were rated as highly important. These increased to a total of 17 components in the third round.

Table 2 provides an overview of the final list of highly rated components of an early supported discharge as identified by the Delphi panellists. The four component groups included one that covered additional/ new staff that would be required in order to run the model. This included a full-time nurse care co-ordinator, and a home visiting podiatrist 1 day per week. In addition, three areas where process changes would be required were identified. The first were changes to the way patients were managed as inpatients, the second was changes to the discharge process, and the final was changes to the way home care was established and managed.

**Table 2:** Final components with ≥80% agreement from Delphi panelists

Component	Specific component	% agreement
New personnel requirements	A full time nurse care co-ordinator	87%
	In home podiatry 1 day per week for wound debridement	87%
Inpatient process components	Identify who needs to be involved in patient’s care on admission and alert the relevant teams	87%
	Early referral to treating team (endocrinology, and seen within 24–48 h	87%
	Shared care between consultants allowing vascular consultants to make decisions or change management as required on other team members behalf	93%
	All lower limb wound patients co-located on the same inpatient ward	87%
	Multidisciplinary ward round including vascular, infectious diseases, endocrinology and allied health representation from dietetics, physiotherapy and occupational therapy	80%
	Orthotist review within 24 h of admission (24 h if not admitted late on Friday)^a^	80%
	Dedicated multidisciplinary foot unit to include input from dietetics, physiotherapy and occupational therapy hours	80%
Discharge process components	Comprehensive management plan in place for patient prior to discharge	100%
	Information provided to GPs around contacts and referral point of access	93%
	Referrals in place for hospital in the home support for patients prior to discharge	86%
	Patients appointed a care-coordinator to make contact prior to discharge to support and co-ordinate discharge process	80%
	Offloading requirements in place prior to discharge	86%
Outpatient process components	Full time nurse care coordinator to act as a single point of contact for patients and GP’s, co-ordinating discharge, following up patients at home and providing reminder for appointments and managing failures to attend	86%
	Appropriate care of the wound in the home in place (could be hospital in the home, district nursing, allied health home visit, or hospital outpatient visits dependent on patient needs and preferences)	86%
Exclusion criteria	Cognitive impairment	93%
	Less than 3 days wait for inpatient angiogram and/or angioplasty	93%

^a^At time of writing, no orthotics was available on weekends

**Fig. 5:**
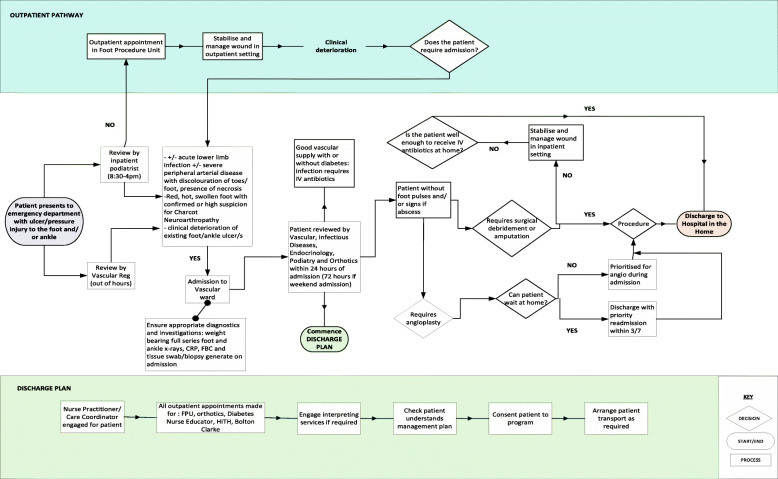
Co-designed Patient Pathway for Early Supported Discharge

Figure 5 provides an overview of the resulting patient pathway that was co-developed from the results of the Delphi. This pathway was established to assist staff with understanding, interpreting and implementing the planned process changes identified in Table 1.

## Discussion

This study identified four key areas for process change required to create an ideal model of ESD: “Inpatient process components”, “Discharge process components”, “Outpatient process components” and “Exclusion criteria”. Across these, 17 key strategies to facilitate an ESD were identified including changes to the way patients are managed in the inpatient wards (both location and timeliness of care by the multidisciplinary team) and the addition of new roles to improve co-ordination and management of the patients once they are at home. Importantly, the co-design process identified that a safe model of ESD requires not only the provision of care in the home that would otherwise have been provided in the hospital, but also a shift to the way care is provided during the hospital stay. To the best of our knowledge, this is the first time a co-design process has been used to develop a model of ESD.

Consistent with other Delphi studies [25–28], we found that a number of components only reached consensus in the third round after review of peer scoring and supporting statements (when the number of agreed upon components almost doubled, from 11 to 19 components). A unique component of this study was the engagement of patients as part of the panel - we found only one other example of people with lived experience participating in a health related Delphi study [29].

A 2017 Cochrane review found that for a number of conditions, ESD improves patient satisfaction and reduces length of stay without increasing the risk of 30-day readmissions [6]. However, this review, and a more recent scoping review of alternative models of service delivery, found no research on ESD models for people with foot disease [6, 30]. Exploring potential ESD models for conditions that are known to require lengthy inpatient stays may benefit not only patients but may improve the sustainability of health systems. A recent Delphi study of national policy makers, health services managers and health services researchers in Australia identified that alternative models of care to support early, safe discharge is a key priority for improving health system sustainability [31].

A strength of this study was the two-phase qualitative process used to both design and then reach consensus on the most important components of an ESD model for foot disease. There are many different approaches to co-design, though all involve engagement of stakeholders using consultative, collaborative or publicly led processes [32]. These processes may include workshops, focus groups, interviews and surveys [32]. To the best of our knowledge, this is the first time a Delphi survey to gain consensus has been used as part of a co-design process and we did find that this method presented some challenges for patient participants. While we found that they engaged easily with the focus group, they had trouble understanding the different components for the ESD model when these were presented in the Delphi. Patient participants were more likely than clinician participants to skip an answer or to change their mind about their rating in the third round following review of median scores. This suggests that the Delphi approach may not be the best method for patient involvement in gaining consensus and other structured facilitation techniques to explore level of consensus, such as nominal group technique [33] or the RAND/ UCLA appropriateness method [34], may have improved patient engagement in the process. An additional limitation of this study is that it is a single hospital study and so the results of the study may not be generalisable to other hospitals or contexts.

The ESD model developed in this study is now in the process of being implemented, and future research in this hospital population will provide insight into whether the model provides care that is as effective and efficient as conventional in-patient hospital care, discharge planning, and post-discharge care. In addition, this research will determine whether the new model can generate meaningful additional impacts, such as reducing length of stay and readmissions, and improving patient and staff experiences across the care continuum. Further research will then be required to identify whether these strategies are relevant to other patient populations.

## Conclusions

Individuals with foot disease often present with a complex medical history that includes frequent hospital admissions and lengthy hospital stays. A model of early supported discharge that would allow these individuals to return home earlier in a way that is safe, acceptable and feasible may result in improving patient satisfaction while reducing health system burden. As this research aimed only to gain consensus on an optimal model of early supported discharge for lower extremity conditions, a future trial and implementation of the model has the potential to make a significant contribution to the experience of care for patients and to the sustainability of the health system.

## Data Availability

The datasets generated and/or analysed during the current study are mostly qualitative in nature and are not publicly available but are available from the corresponding author on reasonable request.

## Abbreviations

QALYs: Quality-adjusted life years
FPU: Foot Procedure Unit
NH: Northern Health
ESD: Early supported discharge

## References

[1] Lazzarini PA, Hurn SE, Kuys SS, Kamp MC, Ng V, Thomas C (2016). Direct inpatient burden caused by foot-related conditions: a multisite point-prevalence study. BMJ Open.

[2] Davis W, Norman P, Bruce D, Davis T (2006). Predictors, consequences and costs of diabetes-related lower extremity amputation complicating type 2 diabetes: the Fremantle diabetes study. Diabetologia..

[3] Bakker K, Apelqvist J, Schaper NC, Board IWGotDFE (2012). Practical guidelines on the management and prevention of the diabetic foot 2011. Diabetes Metab Res Rev.

[4] Holscher CM, Hicks CW, Canner JK, Sherman RL, Malas MB, Black JH (2018). Unplanned 30-day readmission in patients with diabetic foot wounds treated in a multidisciplinary setting. J Vasc Surg.

[5] Cheng Q, Lazzarini PA, Gibb M, Derhy PH, Kinnear EM, Burn E (2017). A cost-effectiveness analysis of optimal care for diabetic foot ulcers in Australia. Int Wound J.

[6] Langhorne P, Baylan S, Trialists ESD. Early supported discharge services for people with acute stroke. Cochrane Database Syst Rev. 2017;7. 10.1002/14651858.CD000443.pub4 PMC6483472 28703869

[7] Echevarria C, Brewin K, Horobin H, Bryant A, Corbett S, Steer J (2016). Early supported discharge/hospital at home for acute exacerbation of chronic obstructive pulmonary disease: a review and meta-analysis. COPD: J Chron Obstruct Pulmon Dis.

[8] Currie C, Tierney A, Closs S, Fairtlough H (1994). Early supported discharge for elderly trauma patients: a report on a preliminary study. Clin Rehabil.

[9] Closs S, Stewart L, Brand E, Currie C (1995). A scheme of early supported discharge for elderly trauma patients: the views of patients, carers and community staff. Br J Occup Ther.

[10] Crotty M, Whitehead CH, Gray S, Finucane PM (2002). Early discharge and home rehabilitation after hip fracture achieves functional improvements: a randomized controlled trial. Clin Rehabil.

[11] Iyengar KP, Nadkarni JB, Ivanovic N, Mahale A (2007). Targeted early rehabilitation at home after total hip and knee joint replacement: does it work?. Disabil Rehabil.

[12] Donetto S, Tsianakas V, Robert G (2014). Using experience-based co-design (EBCD) to improve the quality of healthcare: mapping where we are now and establishing future directions.

[13] Robert G. Participatory action research: using experience-based co-design to improve the quality of healthcare services. Understanding and Using Health Experiences–improving patient care. 2013.

[14] Hasson F, Keeney S, McKenna H (2000). Research guidelines for the Delphi survey technique. J Adv Nurs.

[15] Dalkey NC. The Delphi method. An Experimental Study of Group Opinion. 1967.

[16] Statistics ABo. Socioeconomic Index for Areas http://www.abs.gov.au/websitedbs/censushome.nsf/home/seifa ABS 2016.

[17] Jessup RL, Osborne RH, Beauchamp A, Bourne A, Buchbinder R (2017). Health literacy of recently hospitalised patients: a cross-sectional survey using the health literacy questionnaire (HLQ). BMC Health Serv Res.

[18] Jessup RL, Osborne RH, Beauchamp A, Bourne A, Buchbinder R (2018). Differences in health literacy profiles of patients admitted to a public and a private hospital in Melbourne, Australia. BMC Health Serv Res.

[19] Dillman DA (2007). Mail and internet surveys: the tailored design method.

[20] Walker AM, Selfe J. The Delphi method: a useful tool for the allied health researcher. Br J Ther Rehabil. 1996;3(12):677–81.

[21] Krueger RA (2014). Focus groups: a practical guide for applied research: sage publications.

[22] Glaser BG (1965). The constant comparative method of qualitative analysis. Soc Probl.

[23] Charmaz K (2006). Constructing grounded theory: a practical guide through qualitative analysis: sage.

[24] Heiko A (2012). Consensus measurement in Delphi studies: review and implications for future quality assurance. Technol Forecast Soc Chang.

[25] Vogel C, Zwolinsky S, Griffiths C, Hobbs M, Henderson E, Wilkins E (2019). A Delphi study to build consensus on the definition and use of big data in obesity research. Int J Obes.

[26] Beattie E, Mackway-Jones K (2004). A Delphi study to identify performance indicators for emergency medicine. Emerg Med J.

[27] van Stralen MM, Lechner L, Mudde AN, de Vries H, Bolman C (2010). Determinants of awareness, initiation and maintenance of physical activity among the over-fifties: a Delphi study. Health Educ Res.

[28] Cheung KL, de Ruijter D, Hiligsmann M, Elfeddali I, Hoving C, Evers SM (2017). Exploring consensus on how to measure smoking cessation. A Delphi study. BMC Public Health.

[29] Schneider F, van Osch L, de Vries H (2012). Identifying factors for optimal development of health-related websites: a delphi study among experts and potential future users. J Med Internet Res.

[30] Jessup R, Putrik P, Buchbinder R, Nezon J, Rischin K, Cyril S (2020). Identifying alternative models of healthcare service delivery to inform health system improvement: scoping review of systematic reviews. BMJ Open.

[31] Putrik PJR, Buchbinder R, Glasziou P, Karnon J, O'Connor D. Prioritising models of healthcare service delivery for a more sustainable health system: a Delphi study of Australian health policy, clinical practice and management, academic and consumer stakeholders. Aust Health Rev. 2021; In Press. 10.1071/AH20160 33731250

[32] Slattery P, Saeri AK, Bragge P (2020). Research co-design in health: a rapid overview of reviews. Health Res Policy Syst.

[33] Harvey N, Holmes CA (2012). Nominal group technique: an effective method for obtaining group consensus. Int J Nurs Pract.

[34] Fitch K, Bernstein SJ, Aguilar MD, Burnand B, La Calle (2001). The RAND/UCLA appropriateness method user's manual.

